# The Essential Oil Component Terpinyl Acetate Alters Honey Bee Energy Levels and Foraging Behavior

**DOI:** 10.3390/insects16060561

**Published:** 2025-05-26

**Authors:** Trey Mathews, Ella Joyce, Charles I. Abramson, Harrington Wells, Robert J. Sheaff

**Affiliations:** 1 Department of Biological Science, The University of Tulsa, Tulsa, OK 74104, USAharrington-wells@utulsa.edu (H.W.); 2 Department of Chemical Engineering, The University of Tulsa, Tulsa, OK 74104, USA; emj8494@utulsa.edu; 3 Department of Psychology, Oklahoma State University, Stillwater, OK 74078, USA; charles.abramson@okstate.edu; 4 Department of Chemistry and Biochemistry, The University of Tulsa, Tulsa, OK 74104, USA

**Keywords:** terpinyl acetate, ATP metabolism, essential oil, honey bee, foraging

## Abstract

Essential oils have been utilized in the health, learning/memory, and agricultural fields, but not much is known about the biological activity of their individual components. Terpinyl acetate is a p-menthane monoterpenoid commonly found in cardamom, pine, cajeput, pine needle, and other essential oils. Using a cell culture model system, we found that terpinyl acetate is a potent and specific inhibitor of mitochondrial ATP production, suggesting it might function as a plant toxin. Remarkably, however, terpinyl acetate was not cytotoxic because cells switched to glycolysis to maintain ATP levels. Based on these findings, we hypothesized that terpinyl acetate might be employed to benefit plant survival by modulating metabolism/behavior of plant pollinators such as the honey bee. This hypothesis was tested by investigating terpinyl acetate’s effect on honey bee foraging. Free-flying honey bee flower color choice was recorded when visiting a blue-white dimorphic artificial flower patch of 36 flowers. The nectar–reward difference between flower colors was varied in a manner in which both learning and reversal learning could be measured. Terpinyl acetate ingestion disrupted reversal learning but not initial learning: this change caused bees to remain faithful to a flower color longer than was energetically optimal.

## Simple Summary

Plant essential oils contain many different compounds that facilitate survival, enhance reproduction, and protect from predators. Fractionation of cardamom essential oil identified terpinyl acetate as a biologically active compound that inhibited mitochondrial ATP production, yet was not cytotoxic to tissue culture cells. This result suggests that it was not a plant toxin targeting plant herbivores or pests, but rather served some other function. We hypothesized that terpinyl acetate might be beneficial to plant survival by modulating the metabolism/behavior of plant pollinators such as the honey bee, which led to the present study of testing honey bee foraging decisions on artificial flower patches of blue and white flower. Both initial learning and reversal learning were tested. Terpinyl acetate only disrupted reversal learning performance, causing bees to commit to a flower color despite lower rewards. Such behavioral modification would be beneficial to plant reproduction in the presence of superior competitors.

## 1. Introduction

Essential oils are a natural mixture of volatile compounds extracted from plants, typically by steam distillation [1,2]. They are characterized by unique scents and flavors that have enamored humans since the Middle Ages [3]. Each essential oil contains from a dozen to more than a hundred different compounds, some unique to that plant, while others are more widely present [4,5]. Although some compounds are likely biochemical intermediates, others are end products with specific biological functions [6]. A case in point is terpinyl acetate, a *p*-menthane monoterpenoid commonly found at high concentrations in cardamom, pine, cajeput, pine needle, and other essential oils. Its synthesis is a biochemically complex and energetically expensive process, so from an evolutionary perspective, terpinyl acetate likely has an important biological purpose that is worth the cost [7,8]. That purpose could be protective and modulate the plant itself (e.g., enable resistance to environmental extremes such as temperature), or be protective and target/deter herbivore predators (e.g., insects or herbivores). A third possibility is that terpinyl acetate may have evolved to increase plant fecundity by manipulating pollinator behavior. This latter possibility could be mediated by targeting insect neuronal systems and/or metabolism.

Here, we first utilize a cell culture system to identify terpinyl acetate as an essential oil component with non-lethal biological activity targeting ATP metabolism. Second, based on these results, we go on to test whether terpinyl acetate affects the foraging choices of free-flying honey bees.

Many ancient cultures used essential oils for religious, cosmetology, and medicinal purposes [9,10,11]. Research on essential oil components increased dramatically in recent decades, lending credence to the anecdotal evidence that they exhibit biological activity and may have medicinal uses. In particular, a few essential oils act as antimicrobial agents (reviewed in [5,12,13,14], and some others have significant anti-inflammatory properties (e.g., see: [15,16,17]).

There is also considerable interest in the use of essential oils as insecticides and repellents [18]. For example, the essential oils of both citronella and alfazema are effective in killing fennel aphids. Alfazema is the more effective of the two and has the advantage that it is less damaging to fennel flowers; plus, alfazema does not harm ladybugs, a principal predator of fennel aphids [19]. If fact, there are over 80 plants with essential oils that are now known to deter insects [5] or rodents [20].

Given their extensive use in aromatherapy, the effect of essential oils on neurological systems is also an active area of research. In a meta-review of 70 studies, Sattayakhom identified 14 using non-human animals (rodents, fish, sheep, pig, and chicken), 2 using cell culture, and none involving invertebrates [2]. However, when considering the biological role of a specific chemical in a plant essential oil, insects must be considered since they represent both pollinators [21] and herbivores [22] that every plant must influence.

Honey bees are an excellent model system for evaluating the activity of essential oil components. In their search for nectar and pollen, bees are likely to come in contact with these compounds, which could have evolved to enhance their role as pollinators. Two likely biological targets are the bees’ metabolism, which requires a steady supply of glucose to generate the large amount of ATP needed for flight; and the bees’ behavior, complex behavioral abilities which arise from their sophisticated neuronal circuitry. In fact, there is a diverse and complex range of learning phenomena exhibited by honey bees which was originally thought to be limited to more complex vertebrates [23]. These abilities include categorizing stimuli [24,25], solving delayed matching-to-sample and non-matching-to-sample problems, mastering sameness, identifying the difference in inter-relationships of spatial objects [23], learning contextual information [26,27], categorizing visual information [28,29,30,31], and demonstrating observational learning [32]. Bees can even solve operant conditioning problems [33,34,35,36]. Nevertheless, there are limitations when compared to learning in mammals (e.g., Abramson [37]), which led to an alternative perspective on the learning ability of honey bees (see [38]).

A unique aspect of the experiments reported here is that we use a complex flower patch system that reveals the intricate choices bees make during foraging. These experiments were correlated with an analysis of bee ATP levels to see if terpinyl acetate has the expected metabolic effect as seen in mammalian cell cultures. The flower patch system can also reveal whether the compound reduces discrimination of rewards, as was seen previously in honey bee ethanol studies [39]. Of further importance, the design tests both initial and reversal learning in a discriminant conditioning environment, and thus can reveal alterations in bee behavior beneficial to the plant.

## 2. Materials and Methods

### 2.1. Mammalian Cell Culture ATP Levels

The metabolic effects of terpinyl acetate and 1′8 cineole (another major component of many essential oils) were initially analyzed in the immortalized cancer cell line A549 (immortalized lung adenocarcinoma) obtained from the American Type Culture Collection (ATCC, Manassas, VA, USA). This is a well-characterized cell model for studying inhibition of the various pathways involved in ATP production. Cells were maintained in DMEM media supplemented with 10% fetal bovine serum (FBS, Atlas Biologicals, Fort Collins, CO, USA) in a 37 °C incubator with 5% CO_2_. Cells were switched to Leibovitz-15 (L-15) media, which contains galactose rather than glucose, to analyze compound effects on mitochondrial ATP production. Galactose is shunted into the glycolytic pathway, but this costs 2 ATP, so the net from glycolysis is zero. Thus, pyruvate must proceed to mitochondria and oxidative phosphorylation. If inhibition is specific to mitochondria, then adding glucose to L-15 media will restore ATP levels via glycolysis alone. For experiments in 96-well plates, proliferating cells were removed from the stock plate using trypsin in PBS plus 2.5 mM EDTA. After centrifugation, the cell pellet was resuspended in L-15 media without FBS. The media is buffered with a complement of salts and free-base amino acids in the absence of sodium bicarbonate so that it can be used under conditions of free gaseous exchange with the atmosphere (37 °C, no CO_2_).

Honey bees are multicellular eukaryotes, so at the molecular level, their cell organization and many essential intracellular pathways are similar to those of human cells. This is particularly true of oxidative phosphorylation in mitochondria to produce ATP, one of the most ancient and highly conserved metabolic pathways [40]. In contrast to the large number of available immortalized mammalian cell lines, insect cell lines are much more limited. AmE-711 [41] is the only widely recognized immortalized honey bee cell line, which is derived from embryonic honey bee tissues. This tissue type is more likely to be undifferentiated, which is not the type of cells we would expect to be targeted by terpinyl acetate in the adult bee. Other immortalized insect cell lines that are available (e.g., Sf9, derived from the ovarian cells of the fall armyworm) often grow in suspension and are typically used for biotechnology applications (e.g., protein or virus production). Although our preliminary analysis of terpinyl acetate in A549 cells provided the impetus for subsequent studies, given the results, it will be interesting to investigate terpinyl acetate’s effect on insect cells in the future.

#### 2.1.1. Materials and Supplies

**Figure 1:**
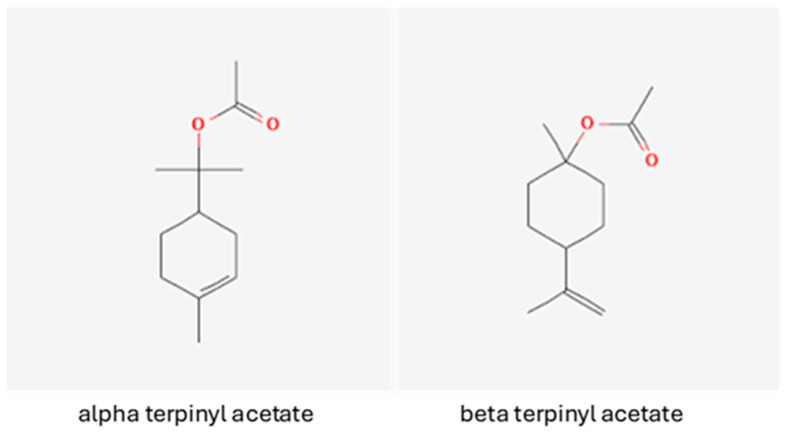
Terpinyl acetate isomer structures (pubchem.ncbi.nlm.nih.gov, accessed on 10 May 2025). Alpha terpinyl acetate is more common in the essential oils.

Pure terpinyl acetate and 1’8 cinoele were obtained from Sigma (St. Louis, MO, USA), and each are a mixture of two isomers: alpha terpinyl acetate and beta terpinyl acetate (Figure 1). The alpha and beta isomers are both esters of terpineol, but the position of the acetate group differs, with alpha terpinyl acetate having its acetate on the opposite side compared to beta-terpinyl acetate (shown in Figure 1). There is approximately 3.5 times more alpha than beta in the pure sample we used. Alpha terpinyl acetate is also more common than β-terpinyl acetate in essential oils such as cardamom and certain eucalyptus varieties. All other materials and supplies were purchased from commercial sources and used without additional purification. 

#### 2.1.2. Terpinyl Acetate Test

To analyze terpinyl acetate’s effect on cell ATP levels, ~20,000 cells were distributed in a 96-well plate containing L-15 media. The terpinyl acetate final concentrations were a 0.976 μM to 1000 μM serial dilution set (diluted in methanol) plus a negative control containing the methanol solvent alone (1% final concentration) (Expt. Group I, treatment 1 through 12). The same set of concentrations were analyzed with glucose (10 mM final concentration) added to the media (L-15 + G) as Expt. Group II, treatments 1 through 12. The plate was incubated at 37 °C, no CO_2_ for 2 h. ATP levels were determined using CellTiterGlo (Promega, Madison, WI, USA), which was added to the 96-well plate following the manufacturer’s instructions. Light emission was quantitated on a Biotek Cytation 5 plate reader (Winooski, VT, USA). ATP levels were normalized across experiments by the number of cells in each well. Each reaction was performed in duplicate. A summary of the terpinyl acetate results is presented in Table 1.

Data are presented as a percentage of the average of the solvent-alone control for statistical analysis following Abebe et al. [42]. Data were analyzed using a 2-way ANOVA (SAS-JMP [43]) with dose, glucose (+ or –), and dose × glucose interaction as factors.

#### 2.1.3. 1′8. Cineole Test

1′8 cineole’s effect on ATP levels used the same protocol described above for terpinyl acetate in L15 ± glucose. Data were again and analyzed using a 2-way ANOVA (SAS-JMP [43]) with dose, glucose (+ or –), and dose × glucose interaction as factors. A summary of the 1′8 cineole cell culture experiments is presented in Table 1.

Rotenone, a well-known inhibitor of complex-1 in the mitochondrial electron transport chain [44], was employed as a positive control to confirm the metabolic pathways involved in ATP production in the cell cultures (2.5 μM final concentration, Table 1). 

### 2.2. Honey Bee Foraging Behavior

Experiments used free-flying honey bees (*Apis mellifera*) foraging on an artificial flower patch. Each trial of an experiment utilized a new set of uncaged free-flying, naïve honey bees (*Apis mellifera*) that had no previous experience with the artificial flower patch. These bees were trained to the flower patch following the methods of Wells et al. (e.g., [45,46,47]). Four or fewer bees were used in each trial of the experiment, with each bee uniquely marked with Testor’s^TM^ enamel paint. Any additional bees that visited the flower patch were removed from the system. Because of differing return trip times, there were only one or two bees on the flower patch at a time, which mimicked a natural foraging environment.

#### 2.2.1. Artificial Flower Patches

Artificial flower patches consisted of 36 flowers spaced 75 mm apart in rows and columns of a 6 × 6 Cartesian coordinate system on a brown pegboard following the design of Wells et al. (e.g., [39,48,49,50,51]). Flowers consisted of a 28 mm × 28 mm plexiglas square, 6 mm thick, with a 5 mm diameter 4 mm-deep well in the center that held the reward. Each flower was mounted on a 90 mm pedicel of 5 mm doweling. The color of each flower was either blue or white. Flower patches consisted of blue and white flowers in equal numbers, randomly arranged with respect to color within the array. Flower color was created by painting the lower surface of each flower blue or white with enamel paint (Testors^TM^ paint Nos. 1208 blue, and 1245 white). The reflectance spectra for the paints and a color hexagon depicting how these colors are perceived by the honey bee can be found in Hill et al. [31].

#### 2.2.2. Experimental Design

Each experiment was initiated by using a new set of bees from an 18-frame hive flying 50 m to a clear Petri dish containing 1 M sucrose solution at the test site.

At the time of an experiment, the Petri dish was removed and replaced with an artificial flower patch. Those bees used in an experiment were each uniquely marked on the thorax with Testors^TM^ enamel paint and defined as naïve to the flower patch. Any additional bees that visited the flower patch were removed permanently from the system.

Each experiment had three treatments given to the bees. Treatment 1 offered foragers 8 μL of 1 M sucrose in both blue and white flowers. Its purpose was to gather baseline data on color choice before bees were given a situation where rewards differed between flower colors. Treatment 2 presented bees with an energy maximization learning experience. It offered foragers the choice of 8 μL of 1.5 M in blue flowers versus 8 μL of 0.5 M sucrose in white flowers. Treatment 3 offered foragers the choice of 8 μL of 1.5 M sucrose in white flowers versus 8 μL of 0.5 M sucrose in blue flowers. Approximately half of the bees were given Treatment 3 before Treatment 2. Thus, the experimental design controlled for flower color preference and tested for initial appetitive learning and for appetitive reversal learning (e.g., Claudio et al. [52]).

At the end of a trial of an experiment, the flower patch was removed and replaced with a clear Petri dish containing 1 M sucrose. All marked bees were terminated. Flowers were washed in unscented detergent, then triple rinsed, and finally allowed to dry after each use, with the flower patch reassembled each day in a novel random arrangement of flowers.

Since the flower patch was reassembled each day in a novel random arrangement of flowers, the spatial arrangement of flowers changed from day to day. It took 4 trials (1 trial per day) with 2 to 4 different bees per trial to obtain data on the 10 to 14 bees used for each terpinyl acetate dose in the experiment.

The use of control group bees also addressed any potential issue with the spatial arrangement of the flowers and the bees’ choices during initial and reversal learning.

As with any experiment, several subjects were used in each experimentally defined group (in our case drug dose), and the experiment took several days (each day with a new set of bees) to gather data for each experimental group. Experiments were all run at the same time of day, in early summer, due to the availability of the investigators (authors). Thus, potential confounding factors should have been the same across all experimentally defined groups.

#### 2.2.3. Terpinyl Acetate Dosing

Bees were captured midway through Treatment 1 upon their return to the flower patch from the hive, immediately after landing on the first flower visited. A captured bee was fed 20 μL 1 M sucrose containing 0, 30, 750, or 10,000 μM terpinyl acetate (from Sigma). Each bee was held for 20 min after consuming the terpinyl acetate solution and then released at the flower patch.

The flower sequence that each bee visited during each trip to the flower patch was recorded. A flower visit was recorded when a bee extended its proboscis into the flower’s well to consume the reward offered and the characteristic abdomen bobbing occurred. The flower’s reward was refilled when the bee made the next flower choice or returned to the hive.

Data were first analyzed using a repeated measure MANOVA (SAS-JMP [43]) with dose, treatment, and dose x treatment as factors. Based on the graphical results, we did the same MANOVA, but only using the 0, 30, and 750 µM dosing groups, which allowed us to combine these groups (lumped groups) for further analysis. Thus, there were now 2 groups: the lumped group and the 10,000 µM dose group. We went on to test for the difference between these two groups at each time-wise stage of the experiment. Three *t*-tests for differences between the means were performed (1—pre-learning, 2—initial learning, and 3—reversal learning) with the Bonferroni correction applied to determine significance level.

### 2.3. ATP Level in Foragers Fed Terpinyl Acetate

Based on the mammalian cell culture results, we were interested in the ATP cellular level in forager honey bees fed terpinyl acetate. Forager ATP level was measured following the methodology of Power et al. [53]. Whole bees were snap-frozen and homogenized in 1 mL 6 M guanidine HCl. Samples were clarified by centrifugation and a portion diluted 1–10 in PBS. Then, 5 μL of the diluted sample was added to 100 microliters of distilled water, and ATP levels were measured using CellTiterGlo (Promega, Madison, WI, USA) according to the manufacturers’ instructions, as was carried out for the mammalian cell culture.

Additionally, the total protein concentration of each sample was determined by the Bradford method (500–0201, Bradford Protein assay kit, Bio-Rad, Hercules, CA, USA). This allowed ATP data to be expressed in terms of the biomass of the sample [54]. Thus, the ATP value was expressed as picomoles ATP per milligram protein (pmol ATP mg^−1^ protein) for whole-bee extractions.

## 3. Results

### 3.1. Mammalian Cell Culture ATP Levels

One of our laboratories (RJS) focuses on identifying and characterizing potential chemotherapeutic agents from natural products such as essential oil. The aim here is to identify compounds that kill cancer cells. In the course of fractionating cardamom essential oil—whose two major components are 1′8 cineole and terpinyl acetate—we identified a biologically active fraction that inhibited mitochondrial ATP production but was not cytotoxic.

**Figure 2:**
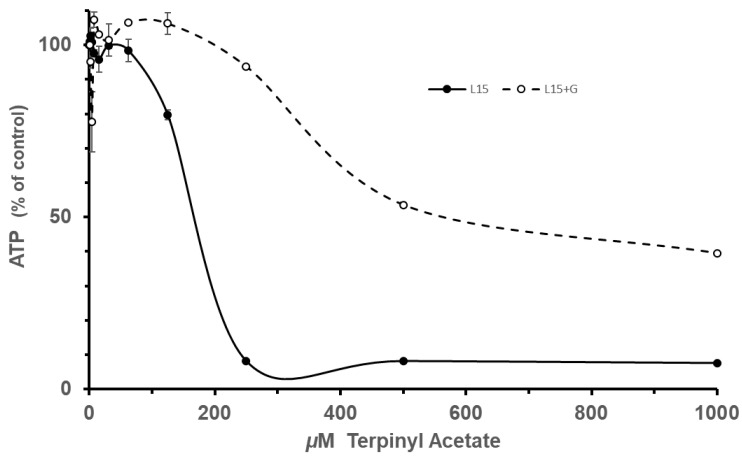
Cell culture ATP levels at different doses of terpinyl acetate. Increasing the dose of terpinyl acetate produced a dramatic reduction in ATP, and the addition of glucose to the media produced a rescuing effect, increasing in ATP.

Terpinyl acetate had a major effect on ATP production by oxidative phosphorylation in the mammalian cell culture experiments (se Expt. Groups I and II in Figure 2). The results of the ANOVA show that dose had a significant effect on ATP level (F_1,1_ = 103.9143, *p* < 0.0001). ATP level dropped to 7.9 ± 0.2 % of the control (mean ± se: combining 500 and 1000 dose ATP levels base on Figure 1). In addition, glucose (+ or −) had a significant effect on ATP level (F_1,1_ = 9.2248, *p* = 0.0040) in the presence of terpinyl acetate. Adding glucose completely restored ATP levels at low terpinyl acetate concentrations (~250 micromolar) and slowed the drop in ATP level at higher terpinyl acetate concentrations (Figure 2). Finally, we observed a glucose × dose interaction effect (F_1,1_ = 7.8857, *p* < 0.0074). These effects were reflected in a change in the IC_50_ values_,_ a measure of drug potency in pharmacology. Using AAT Bioquest, the IC_50_ for terpinyl acetate in L-15 media was 143 micromolar, while the value in L15 + glucose rose to 397 micromolar [55]. These results are consistent with terpinyl acetate specifically targeting ATP production by oxidative phosphorylation on the mitochondria. 

**Figure 3:**
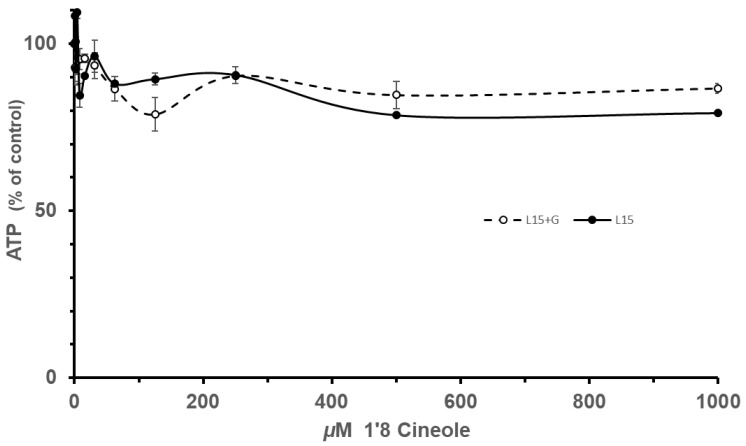
Cell culture ATP levels at different doses of 1′8 cineole. The addition of glucose to the media did not produce a significant increase in ATP, and increasing doses of 1′8 cineole produced a minimal decrease in cell culture ATP level.

In contrast, the mammalian cell culture experiments with 1′8 cineole (Expt. Groups III and IV) demonstrated that it had little effect on ATP level (Figure 3). Neither the glucose (+ or −) effect (F_1,1_ = 0.0770, *p* = 0.7827) nor the interaction of the glucose × dose effect (F_1,1_ = 1.7429, *p* = 0.1936) were significant (ANOVA). However, the 1′8 cineole dose effect was significant (F_1,1_ = 16.4353, *p* = 0.0002) but slight in magnitude (Figure 3).

Since neither a glucose nor an interaction effect was observed, the ATP levels for + and—glucose were lumped along with the 500 and 1000 level doses (Figure 2) to give a measure of the ATP reduction by 1′8 cineole. The ATP level was 82.1 ± 1.5% (mean ± se) of the control. The lack of a glucose effect and only a slight reduction in ATP level suggests that 1′8 cineole is not a mitochondrial inhibitor, and the effects observed were likely due to a mild, general cellular toxicity.

Expt. Group V represents a cell culture positive experimental control. Rotenone is a potent inhibitor of complex 1 of the electron transport chain. When rotenone was added to the L15 culture media lacking glucose, ATP levels were depressed 78% (without rotenone 100.00 ± 1.86% *n* = 6 vs. with rotenone 22.85 ± 1.18% *n* = 6, mean ± se). Adding glucose completely overcame rotenone inhibition as the cells switched to ATP production via glycolysis using the well-known Warburg effect (without rotenone 100.00 ± 1.17% *n* = 6 vs. with rotenone 96.71 ± 1.03% *n* = 6, mean ± se) [56].

### 3.2. Honey Bee Foraging Behavior

Foragers experienced three treatments when visiting a flower patch. Treatment 1 always came first and offered 1 M sucrose reward in both flower colors. Treatment 2 offered the foragers 1.5 M sucrose in blue flowers and 0.5 M sucrose in white flowers, whereas Treatment 3 offered bees 0.5 M sucrose in blue and 1.5 M sucrose in white flowers. For half of the bees, Treatment 3 came before Treatment 2 to control for treatment time-wise order. Results of the repeated measures MANOVA is where the significant treatment (F_2,41_ = 110.66, *p* < 0.0001) and treatment x dose (F_2,41_ = 7.00, *p* = 0.0024) effects occurred, but the dose effect alone (F_1,42_ = 0.47, *p* = 0.496) was not significant.

**Figure 4:**
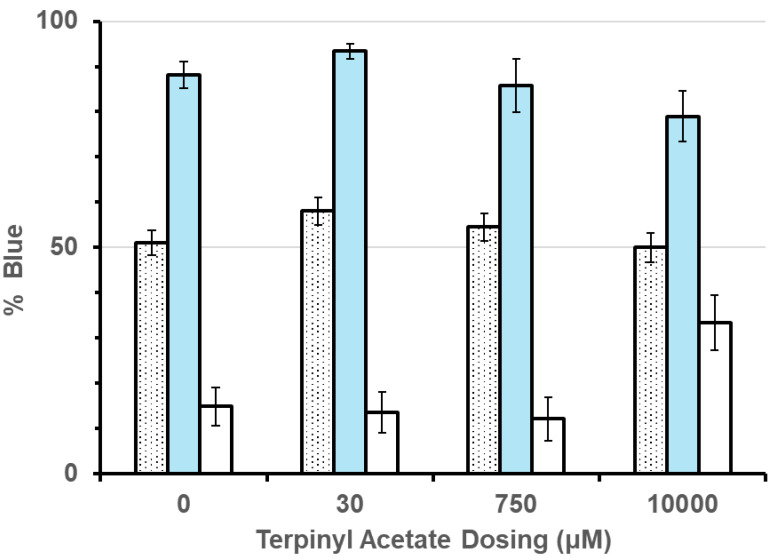
Free-flying bees visited flower patches of 18 blue and 18 white flowers. Treatment 1 had 1 M sucrose in both blue and white flowers (dotted bars). Treatment 2 had 1.5 M sucrose in blue and 0.5 M sucrose in white (blue bars). Treatment 3 reversed the rewards having 0.5 M sucrose in blue and 1.5 M sucrose in white flowers (white bars).

A treatment effect was predicted by the experimental design if initial learning and/or reversal learning occurred. Based on energy maximization models, bees should not favor either flower color in Treatment 1, favor blue flowers in Treatment 2, and favor white flowers in Treatment 3. This can clearly be seen to occur in Figure 4.

The lack of a dose effect uniformly across treatment is also apparent in the data (Figure 4), regardless of what terpinyl acetate dose bees favored for blue flowers in Treatment 2 and white flowers in Treatment 3. The lack of a stand-alone dose effect is also not surprising since Treatment 1 should result in random flower color selection whether or not flower color selection followed energy maximization or a drug induced lack-of-learning model, whereas Treatment 2 and 3 flower selection should be dose-dependent.

The treatment x dose effect shows that behavior is modified by the terpinyl acetate dose, affecting the percent of blue flowers visited depending on the flower color offering the better reward in conjunction with a dose effect.

We went on to test whether dose, treatment, and/or dose × treatment were significant effects using only the data of 0, 30, and 750 μM doses (lowest three doses). Treatment was a significant effect (F_2,26_ = 113.91, *p* < 0.0001), but not dose (F_1,27_ = 1.56, *p* = 0.227) nor treatment × dose (F_2,26_ = 0.37, *p* = 0.697). Thus, a behavioral effect of terpinyl acetate was only seen in the 10,000 μM dose (10 mM), and thus for further analysis, the 0, 30, and 750 μM doses can be lumped.

**Figure 5:**
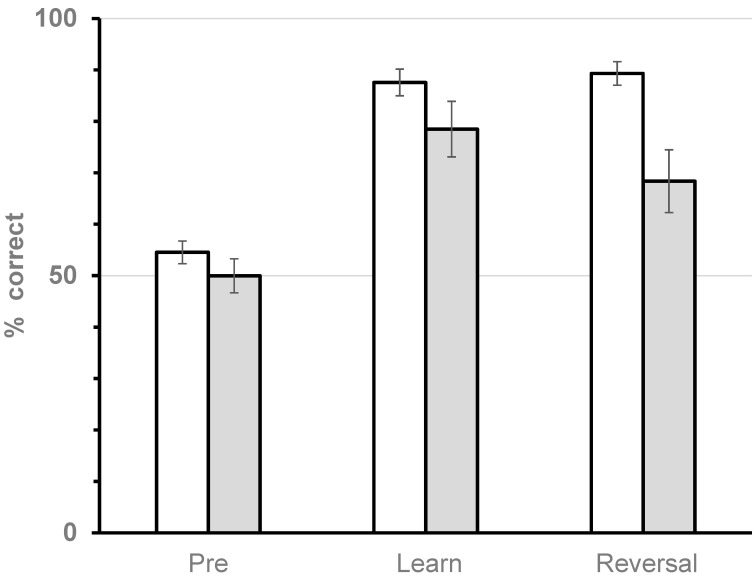
Free-flying honey bees visited flower patches of 18 blue and 18 white flowers. Time order represents pre-learning (treatment 1: gray 1 M and white 1 M sucrose), initial learning, where half of the time treatment 3 (gray 0.5 M and white 1.5 M sucrose) came before Treatment 2 (gray 1.5 M and white 0.5 M sucrose), and reversal learning, which occurred last. White bars are the 0, 30, and 750 μM data lumped because there was not a significant ANOVA result, and grey bars are the 10,000 μM dose. Difference exists only in reversal learning.

We were interested in whether terpinyl acetate affected both initial learning and reversal learning. To test this model, the data were divided into two groups based on the above repeated measures MANOVA analyses. Foragers receiving the 0, 30, or 750 μM doses were combined into a control group. Bees receiving the 10,000 μM dose were in the second group (experimental). Furthermore, the data were divided into pre-learning (i.e., Treatment 1), initial learning (the second time-wise treatment, regardless of whether blue or white flowers offered the better reward), and reversal learning (the third time-wise treatment, again regardless of whether blue or white flowers offered the better reward). We examined the percent correct (visit to the higher rewarding flower color in the initial and reversal learning scenarios, and percent blue flowers visited in the pre-learning group (flowers had equal rewards). The data are displayed in Figure 5. *t*-tests were used to compare the control to the experimental group in each of the three situations (pre, initial, and reversal) separately.

The results of the *t*-tests with Bonferroni correction are that a significant difference did not occur between the control and experimental groups in either the pre-learning comparison (t_42_ = 1.353, *p* = 0.549) or initial learning comparison (t_42_ = 1.504, *p* = 0.42). However, the control and experimental groups were significantly different in the reversal learning comparison (t_42_ = 4.081, *p* = 0.0006); reversal learning deteriorated in bees fed the 10,000 μM dose, as can clearly be seen in Figure 5.

### 3.3. ATP Level in Foragers Fed Terpinyl Acetate

**Figure 6:**
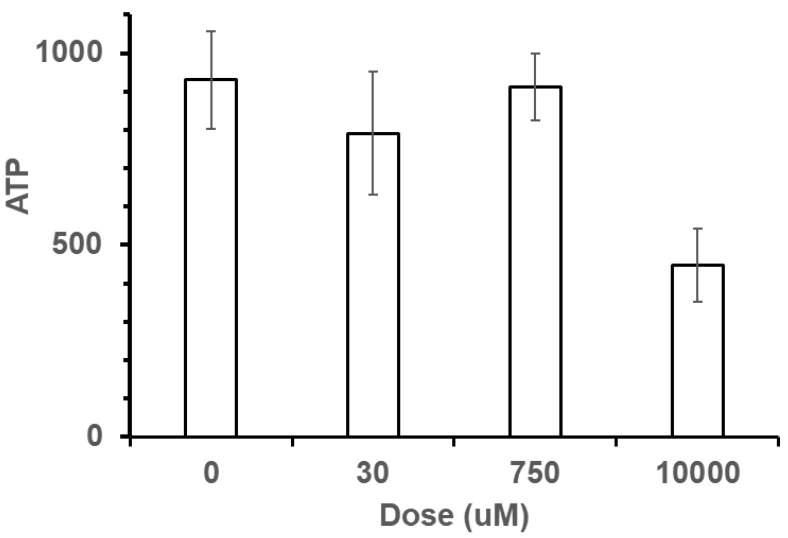
ATP level in bees fed 20 μL of terpinyl acetate of differing dosage after being held for 20 min. ATP levels are depressed for the 10,000 μM terpinyl acetate dose.

ATP levels were measured in foragers from each terpinyl acetate dose: 0, 30, 750, and 10,000 μM. The data were analyzed using a one-way ANOVA (Figure 6). A significant difference existed among groups (F_1,38_ = 8.4579, *p* = 0.0060). However, when the same analysis was performed using only bees from the 0, 30, and 750 μM terpinyl acetate doses, a significant difference did not occur among groups (F_1,28_ = 0.0787, *p* = 0.7811). The fall in ATP level closely followed the foraging behavior results. Only in the 10,000 μM-dosed bees was there a significant drop in ATP level.

## 4. Discussion

### 4.1. Cell Culture

Our cell culture results reveal some novel findings. First, terpinyl acetate is a potent and specific inhibitor of mitochondrial ATP production. Doses as low as 200 μM terpinyl acetate reduced cellular ATP levels by over 90% of that observed in the control group. The fact that the addition of glucose restored ATP levels indicates that terpinyl acetate is specifically targeting oxidative phosphorylation rather than blocking glycolysis. These results also rule out an indirect effect of terpinyl acetate reducing cell viability., In marked contrast, the other major component of cardamom essential oil, 1′8 cineole, did not affect cellular ATP levels. Terpinyl acetate is a relatively simple nonpolar molecule, and we have not observed any extensive specificity of terpinyl acetate activity in different cell lines. Together, these results suggest that terpinyl acetate readily crosses cell membranes, and that it is targeting a biological component/system common to all cells and involved in mitochondrial ATP production. The most likely candidates are the tricarboxylic acid (TCA) and electron transport chain (ETC), both of which are located in the mitochondria of eukaryotic cells.

The hypothesis that terpinyl acetate inhibits TCA or ETC function is supported by the following: (1) the observation that terpinyl acetate inhibits ATP production in L15 media without glucose (forces cells to use galactose, which must go through oxidative phosphorylation); and (2) glucose addition overcomes terpinyl acetate inhibition by allowing cells to produce ATP via glycolysis. These results indicate that terpinyl acetate is not targeting galactose metabolism, but rather something in common with the galactose and amino acid production of ATP. Again, the common denominator is mitochondrial TCA and ETC. Work is currently underway to identify the specific mitochondrial target, but presently, we have only noteworthy speculations on structural similarities observed between terpinyl acetate and ubquinone, an important component of the ETC. The 1′8 cineole, which did not inhibit oxidative phosphorylation, lacks these structural elements.

### 4.2. Foraging Behavior

Based on the cell culture experimental results, we suspected that terpinyl acetate would greatly tax the cellular energy levels of insects such as the honey bee forager. Indeed, we expected forager activity to cease, and foragers to fail to return to the flower patch. Instead, foragers readily returned to the flower patch repeatedly regardless of the terpinyl acetate dose, even when given 20 μL of 10,000 μM terpinyl acetate. In fact, only 1 bee out of 45 in the entire set of experiments failed to return, and it was not from the highest terpinyl acetate group. Terpinyl acetate thus does not appear to function as a pollinator deterrent.

There was also a surprising result in terms of forager learning. At our highest dose of terpinyl acetate, reversal learning was retarded, but not initial learning. Reversal learning is an experimental design to evaluate an organism’s ability to adjust to changing reward conditions [57] and has become an important technique for evaluating behavioral adjustment to environmental change in honey bees as well as many vertebrate species.

In honey bees, reversal learning has been used, for example, to distinguish differences among subspecies [52], castes [58], and among scout and non-scout bees [59]. Reversal learning is also used to assess memory [60], and the effect of ethanol impaired leaning in bees [61]. Although reversal learning has been used to detect differences in learning among honey bee subspecies, this is the first time that an essential oil has been found to disrupt reversal learning performance [52,61,62]. The results have similarities to what was reported for honey bee proboscis extension response conditioning (PER) when foragers were used as an EtOH model. Reversal learning was slowed compared to initial learning. However, there is a difference from terpinyl acetate’s effect in that ethanol also lowered how well bees eventually became in both initial and reversal learning [61]. In terms of humans, the reversal learning paradigm is now proving to be important in medical diagnostics of diseases such as psychopathy, Parkinson’s, schizophrenia, and developmental delays, which makes the underpinnings of the behavior a particularly ‘hot topic’, but still not well understood [57].

In vertebrates, the foundations for reversal learning appear quite complex, and the literature is not completely consistent in terms of conclusions [57]. Nevertheless, there appears to be a neuron-specific component to reversal learning as shown by excitotoxic lesions in the orbitofrontal cortex of rodents that impair reversal learning but spare initial learning (e.g., Diaz et al. [63]). In addition, there is a neurotransmitter component to reversal learning that involves not only the neurotransmitter, but also receptor types. For example, serotonin seems to facilitate reversal learning, but this appears to have region specificity (e.g., cortex) and a receptor-type component (e.g., Nilsson et al. [64]). Dopamine and its D2 receptor also play a role in reversal learning in vertebrates. The activation of the D2 receptor coupled with weaker dopamine signaling seem to characterize reversal learning scenarios (e.g., Calabresi et al. [65]). On the other hand, glutamate’s role is less clear in reversal learning, with the many apparently conflicting studies leading Izquierdo [57] to conclude that brain region may be important in defining glutamate’s roles in discrimination and reversal learning tasks. Although vertebrates present a model in part for honey bees, the difference alone in brain structure as well as the component of stimulus removal perception differences between honey bees and vertebrates suggests that there will be some important differences [37,38].

### 4.3. The Floral Marketplace

Ecology and evolution go hand in hand. While it is tempting to consider spatial and temporal floral diversity from the perspective of the pollinator, it is just as important to consider competition in this respect to issues involving plant niche overlap in terms of pollination.

Competition is one of the driving forces of evolution, and as such, has long been known to be important in defining the distribution of plant species [66]. While much attention has been paid to plant niche overlap and the resultant competition for water, light, and soil nutrients (e.g., Craine and Dybzinki [67]), evolutionary fitness depends on both survival and fecundity, being the mathematical product of the two factors. Thus, even when survival is superb, fecundity issues can drive a population to near extinction.

In particular, diminished animal populations are known to become endangered due to the Allele effect [68], whereby mate location becomes problematic due to dispersion of individuals (e.g., Wells et al. [69]). Flowering plant species face the fecundity issue as well, even when niche overlap for abiotic factors does not occur. While we may view an ecological setting as a “floral market” from which foraging bees make choices that ultimately relate to colony wellbeing, this “marketplace” importantly represents intense competition between angiosperm species for pollination that ultimately determines plant species fecundity (e.g., Randall et al. [70]).

Floral landscapes change over time, and from the evolutionary view of an insect pollinator, energy maximization principles should prevail in flower species choice (see [46,71,72]). Change in floral composition in the foraging environment is not only seasonal, but also diurnal due to the endogenous rhythms of angiosperms [73,74,75]. In a simplistic view, a forager should focus on net calories per unit time, but estimation can be quite complex when considering travel time, handling time, variation in reward quality, variation in reward quantity, the magnitude of the resource, and even competitors for nectar.

From the plant’s perspective, maintaining reward superiority for pollinators is a losing proposition during the endogenous and seasonal flowering rhythms of plants in a community. This conclusion follows from the marginal value theorem that posits that predators (here insect nectivores) should abandon patches of prey (here a flower species) well before they are depleted [76]. The result can be a significant difference between the potential fecundity of a plant species and the realized fecundity due to pollinators switching to a newly emerging nectar resource (abandoning the “patch”); theoretically, the drop in fecundity could even be disastrous in terms of a plant species’ fitness.

Our results suggest that some plant species may have found a partial solution to this fitness dilemma in terpinyl acetate as a component of its essential oil. Results of this study suggest that, in nature, honey bee foragers would remain loyal to a ‘terpinyl acetate’ plant species past when the marginal value theorem predicts the plant species should be abandoned in favor of a competitor’s flowers. There are a couple of scenarios where this seems beneficial for the plant: (1) when a highly rewarding plant taxa comes into flower midway through the flowering season of the ‘terpinyl acetate’ plant species, and (2) as the flowering season of the ‘terpinyl acetate’ plant species wanes and bees continue to visit the remaining flowers. Terpinyl acetate may act as a plant’s ‘loyalty drug’ in terms of bee foragers. However, honey bees are just one of a great variety of insect pollinators, and with diversity undoubtedly there will be differences.

### 4.4. Insect Diversity

The contrast between the mammalian cell culture and free-flying honey bees, the experiments seen here suggest that there is much more evolutionary fine tuning of the essential oil than a gross cellular overview would predict. In fact, this disparity suggests that an essential oil component may affect insects of highly divergent taxa in different ways or at least to different extents. Just such a scenario is seen with the essential oils of fennel and pignut.

In Brazil, the essential oils of sweet fennel and pignut are used as a natural pesticide to control aphids on crop plants. However, much higher concentrations of this biocide are needed to affect the mortality of the Africanized honey bee than aphids [77]. The same type of situation occurs with fennel aphids and ladybugs when using the essential oil alfazema as a biocide. Ladybugs are not affected by alfazema essential oil concentrations used to control fennel aphids [19].

As we show with terpinyl acetate, the effect of an essential oil may be more targeted at behavioral modification rather than mortality. This seems to also be the case with honey bee exposure to the pesticide Bioganic^®^, which is composed of the essential oils of thyme, clove, sesame, and wintergreen. Bioganic^®^ is not toxic per se to honey bees at the doses used; foragers do not die or go into a stupor. In fact, they continue to feed. Nevertheless, this pesticide is deleterious in that it affects forager learning. Pavlovian conditioning, as seen in the proboscis extension reflex in both simple and complex learning situations, becomes slower and less complete [78].

Of particular interest is the fact that the odors of essential oil biocides are not even repellent to honey bees. For example, foragers will readily use the fennel–pignut oil scent as a conditioning cue to recognize nectar rewards [78]. The same is true for citronella. Contrary to the anecdotal evidence that the essential oil of citronella is repellent to honey bees, Abramson et al. [79] showed that the repellent effect is based on a change in the stimulus conditions, which confuses the honey bee temporarily and is not the effect of citronella per se as a repellent.

Taking the above observations into account, we would project a difference in the effect/effectiveness of terpinyl acetate on the behavioral ecology of colonial insect foragers from those that are solitary insects.

For colonial insect pollinators which provision the colony (e.g., bumble bees), and probably for solitary bees that provision each larva cell (e.g., carpenter bees), as a working hypothesis, we would expect to observe what we saw with honey bees; here, terpinyl acetate acts to keep foragers loyal to the plant species as flowering wanes. However, this is just speculation and is a rich area for study. In fact, there may well be physiological differences among insect pollinators in response to terpinyl acetate as has been observed in the use of insect models to study EtOH drug effects (e.g., Brogna et al. [80]).

In stark contrast to honey bees, insect pollinators (such as lepidoptera) that do not store nectar would become satiated and quit foraging for some period of time. Here, terpinyl acetate may not act as a “loyalty” drug. Rather, as one of the authors (RJS) initially proposed, terpinyl acetate may act as a “feedent”. Decreased catabolism efficiency in producing ATP could result in metabolic compensation whereby glycolysis is increased. Indeed, we see mammalian cancer cells depend totally on glycolysis if not forced to use the TCA cycle of the mitochondria. This metabolic switching process possibly could occur in insect “sugarvores” when forced to use glycolysis by compounds such as terpinyl acetate. The behavioral impact for this type of insect pollinator would be metabolizing sugar at a significantly increased rate through glycolysis and hence foraging more often for possibly longer periods of time. This would be an alternate scenario for plant manipulation of insect pollinators by terpinyl acetate; one that also needs to be experimentally explored.

## 5. Conclusions

Terpinyl acetate appears to have a very different ecological role in the cardamom essential oil than does the oil’s major component, 1′8 cineole. At this point in our research, we can only speculate on the specific mechanism of terpinyl acetate’s mode of action. One suggestion is that terpinyl does not affect simple discrimination, but when the situation is made more complex by switching the contingencies, bees initially have problems adjusting. A second interpretation is that the effect of terpinyl acetate takes time and that this carries over into the reversal phase. In either case, the desired effect for the plant is accomplished.

Terpinyl acetate’s effect is likely from chronic exposure over the course of a day. Cardamom essential oil is extracted from dried seeds, which contain 4 to 8% essential oil. The major components of the extracted essential oil are 1,8 cineole (~55%) and terpinyl acetate (~29%) [81]. While terpinyl acetate concentrations in cardamon nectar and pollen are not known, a study of thyme (which also contains terpinyl acetate) found that it was present in nectar at 1.69 ppm, which translates to 8.6 micromolar [82]. Chronic versus a single dose exposure would be another important area of study, not only for terpinyl acetate, but also for the components of essential oils in general. 

The broader implications are that we should not be talking about the benefits/harms of a particular essential oil, but rather about each component’s action. Indeed, in many cases, we may need to consider not only the general biological activity, but also the behavioral ecology of involved animal species and the evolutionary purpose. As we have shown disruption in the reversal learning of free-flying foragers, it would be of interest to see if a similar result is found in other honey bee learning paradigms such as proboscis extension conditioning [83], aversive conditioning (e.g., [62]), and operant conditioning [33,35,36,84]. We see our work as a first step in understanding how terpinyl acetate leads alterations in bee behavior, so our study has its limitations. Corroboration is needed using a natural floral landscape with bees as well as other pollinators such as Lepidoptera. Hemolymph levels of terpinyl acetate that produce the behavioral results seen here need to be determined, and the exact mechanism by which this drug alters bee physiology remains to be determined. Nevertheless, results herein provide important new insights into the complex interplay between plants and their honey bee pollinators.

## Figures and Tables

**Table 1:** Cell culture experiments. A summary of the experiment performed with mammalian cell cultures. Treatment 1 is a negative control in each serial dilution Expt. Groups I to 4. Expt. Group V is a positive control.

Experiment			Cell Growth Media			Compound			Treatments		
Expt Group I			L-15			Terpinyl acetate			1 through 12		
Expt Group II			L-15 + 10 mM glucose			Terpinyl acetate			1 through 12		
Expt Group III			L-15			1’8 Cineole			1 through 12		
Expt Group IV			L-15 + 10 mM glucose			1’8 Cineole			1 through 12		
Expt Group V control			L-15 ± 10 mM glucose			Rotenone			±2.5 μM		
**Treatments 1 through 12: Compound μM**											
#1	#2	#3	#4	#5	#6	#7	#8	#9	#10	#11	#12
0.0	0.98	1.95	3.91	7.81	15.6	31.3	62.5	125	250	500	1000

## Data Availability

The original contributions presented in this study are included in the article. Further inquiries can be directed to the corresponding author.
